# A neonicotinoid impairs olfactory learning in Asian honey bees (*Apis cerana*) exposed as larvae or as adults

**DOI:** 10.1038/srep10989

**Published:** 2015-06-18

**Authors:** Ken Tan, Weiwen Chen, Shihao Dong, Xiwen Liu, Yuchong Wang, James C. Nieh

**Affiliations:** 1 Key Laboratory of Tropical Forest Ecology, Xishuangbanna Tropical Botanical Garden, Chinese Academy of Science, Kunming, Yunnan Province, 650223 China; 2 Eastern Bee Research Institute, Yunnan Agricultural University, Heilongtan, Kunming, Yunnan Province, 650223 China; 3 Division of Biological Sciences, Section of Ecology, Behavior, and Evolution, University of California, San Diego La Jolla, California, USA

## Abstract

Xenobiotics such as the neonicotinoid pesticide, imidacloprid, are used globally, but their effects on native bee species are poorly understood. We studied the effects of sublethal doses of imidacloprid on olfactory learning in the native honey bee species, *Apis cerana*, an important pollinator of agricultural and native plants throughout Asia. We provide the first evidence that imidacloprid can impair learning in *A. cerana* workers exposed as adults or as larvae. Adults that ingested a single imidacloprid dose as low as 0.1 ng/bee had significantly reduced olfactory learning acquisition, which was 1.6-fold higher in control bees. Longer-term learning (1-17 h after the last learning trial) was also impaired. Bees exposed as larvae to a total dose of 0.24 ng/bee did not have reduced survival to adulthood. However, these larval-treated bees had significantly impaired olfactory learning when tested as adults: control bees exhibited up to 4.8-fold better short-term learning acquisition, though longer-term learning was not affected. Thus, sublethal cognitive deficits elicited by neonicotinoids on a broad range of native bee species deserve further study.

Bees are significant pollinators of natural ecosystems and agricultural crops^1^,^2^. Impairment of bee foraging abilities or reductions in colony health should therefore negatively affect the key pollination services that they provide^3^,^4^. Xenobiotics, foreign substances that include man-made chemicals such as pesticides, can negatively affect bee foraging and pollination, reducing colony fitness^5^ and contributing to bee population declines^6^. Neonicotinoid pesticides have received particular attention because they are widely used^7^ and disrupt bee foraging in multiple ways, even at sublethal doses^8^. Research on neonicotinoid sublethal effects is crucial because assays that only test for lethality do not reveal more subtle impairments such as degraded bee learning, which can affect colony health and foraging and pollination^8^,^9^,^10^. In addition, most studies examining pesticide effects on bees have focused on a single honey bee species, *A. mellifera*, even though other honey bee species also play an important role in ecosystem and crop pollination^11^,^12^, can be more sensitive than *A. mellifera* to certain pesticides^13^,^14^ and contribute to pollination services that are vital for human nutrition^15^.

We therefore studied the effects of a neonicotinoid pesticide, imidacloprid, that is widely used throughout China^16^ on a native honey bee species, *A. cerana*, which is an important pollinator of agricultural^17^,^18^ and native Asian plants^18^,^19^. In China alone, more than two million managed colonies of *A. cerana* are used for honey production and crop pollination^18^. *Apis cerana* occurs throughout southern and eastern Asia, with a geographic range extending from India to China^20^.

Imidacloprid is a systemic insecticide that is readily absorbed by plant tissues and is found in nectar and pollen consumed by bees^21^. It can linger in soil, leach into groundwater, and be incorporated in plants that were not initially treated^22^. Moreover, imidacloprid degradation from environmental decay and insect metabolism yields products that are also toxic to bees^21^. Imidacloprid and its metabolites act by binding to nicotinic acetylcholine receptors (nAChR) on honey bee neurons^23^. In *A. cerana*, imidacloprid binds to a nAChR receptor^24^. Imidacloprid consequently exerts a broad suite of sublethal neural effects: brain cell death^25^, impaired motor function^26^,^27^, reduced food uptake^28^, decreased foraging^29^, diminished hive entrance activity^30^, reduced predator avoidance^31^, impaired navigation back to the nest^32^,^33^, and compromised learning^34^.

Olfactory learning allows bees to associate floral odors with nectar rewards and thereby facilitates foraging^35^ and floral constancy (important for efficient pollination^36^). In *A. mellifera*, sublethal doses of imidacloprid^30^,^37^,^38^ or a primary metabolic byproduct (5-OH imidacloprid)^39^ significantly impair short-term and longer-term olfactory learning^34^. Some studies suggest that xenobiotics can be harmful to *A. cerana* cognitive processes. *Apis cerana* foragers feeding on sugar solution with imidacloprid at 40 μg/L (<0.52 ng/bee) had impaired decision-making and did not avoid nectar with a dangerous hornet predator, unlike controls^31^. Flumethrin, a pyrethroid commonly used to kill *Varroa* mites in honey bee colonies, interferes with *A. cerana* olfactory learning^40^. However, no studies to date have tested if neonicotinoids can impair associative learning in *A. cerana*.

Recently, Yang *et al.*^38^ showed that honey bees (*A. mellifera*) exposed as larvae, even to very small doses of imidacloprid, had impaired olfactory learning as adults. A very low dose of 0.04 ng/larvae significantly reduced subsequent adult learning by 58%-63% in comparison with control bees. Larvae can be exposed to such xenobiotics through rearing in combs contaminated with pesticide residues, and consequently suffer higher brood mortality and reduced adult lifespan^41^. It is unknown if imidacloprid will similarly affect *A. cerana* larvae. We therefore tested the sublethal effects of imidacloprid on olfactory learning when bees were exposed as adults or as larvae Fig. 1 and also tested the effects of imidacloprid treatment on successful larval development (measured as cell capping) and survival to adulthood.

## Results

### Experiment 1: adult exposure

#### Short-term memory

**Figure 1:**
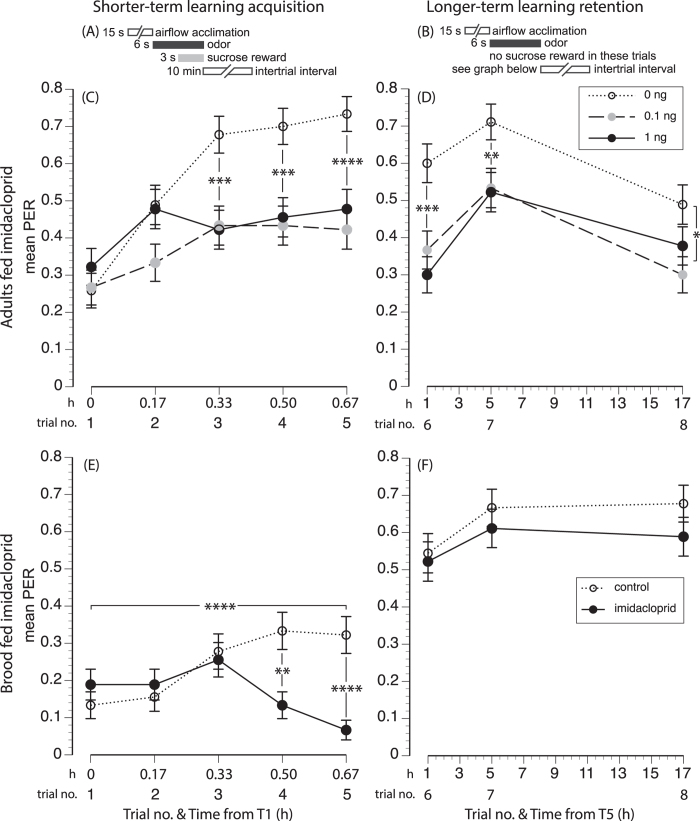
Effect of imidacloprid on olfactory PER learning in *A. cerana* bees treated when they were adults or brood. The temporal design of the (**a**) short-term and (**b**) longer-term trials is shown. For bees treated and tested as adults, we show mean PER for (**c**) short-term learning acquisition (elapsed time from first trial shown) and (**d**) longer-term learning retention (elapsed time from the last reinforced learning trial, t5, shown). For bees treated when they were larvae and tested as adults, we show mean PER for (**e**) short-term and (**f**) longer-term learning. Lines link points with significant contrasts (*P < 0.05, **P ≤ 0.01, ***P ≤ 0.001, ****P ≤ 0.0001). Standard error bars are shown. The x-axes show time (h) and trial numbers.

Control bees fed imidacloprid as adults exhibited short-term learning that improved with reinforcement (trial effect: *F*_4,1245_ = 16.27, *P* < 0.0001). However, both imidacloprid treatments impaired short-term learning. For trials 3-5 (t3-5), control group learning was significantly higher by 1.6-fold than in the pesticide groups (LSM contrasts _t3, t4, or t5_: *F*_1,339_ ≥ 12.35, *P* ≤ 0.0005). There was no significant overall effect of treatment (*F*_2,341_ = 0.36, *P* = 0.70), but there was a significant interaction of treatment*trial (*F*_8,1245_ = 3.34, *P* = 0.0008) because learning curves of imidacloprid-treated bees had significantly different slopes than learning curves of control bees (Fig. 1C). Colony and individual bee identity respectively accounted for 0.7% and 15.7% of model variance.

Longer-term memory retention (t6) decreased slightly relative to short-term memory (t5) in control bees (1.2-fold higher in controls, LSM contrast _t5 vs. t6_: *F*_1,2046_ = 4.11, *P* = 0.04) and imidacloprid-treated bees (1.4-fold higher in controls, LSM contrast _t5 vs. t6_: *F*_1,2046_ = 6.29, *P* = 0.01, data from both imidacloprid concentrations pooled, Fig. 1C–D).

### Longer-term memory

Control-treated adult bees exhibited significantly better longer-term memory than imidacloprid-treated bees (treatment effect: *F*_2,87_ = 6.44, *P* = 0.003). Control group bees exhibited significantly higher longer-term memory by 1.3-1.8 fold than pesticide-treated bees (LSM contrasts _t6 and t7_: *F*_1,236_ ≥ 6.39, *P* ≤ 0.01; LSM contrast _t8_: *F*_1,236_ ≥ 4.28, *P* = 0.04, Fig. 1D).

Memory retention changed over time (trial effect, *F*_2,716_ = 15.96, *P* < 0.0001): memory was poorer at 1 h and 17 h than at 5 h (Fig. 1D). However, memory at 1 h vs 17 h was not significantly different (LSM contrast_t6 vs t8_ : *F*_1,712_ = 0.77, *P* = 0.38). The rate of memory extinction was not affected by treatment: there was no significant interaction of treatment*trial (*F*_4,712_ = 1.17, *P* = 0.32). Colony and bee identity respectively accounted for 0.1% and 17.2% of model variance.

### Experiment 2: brood exposure

#### Brood survival

Larvae were fed a daily dose of 0.04 ng dose/bee, repeated for 6 days, resulting in a total dose of 0.24 ng/bee. There was no significant effect of larval treatment on the number of sealed cells or the number of bees that emerged (*χ*^2^_1_ ≤ 0.27, *P* ≥ 0.60). On average, 91.0 ± 2.6% and 85.7 ± 1.5% of control and imidacloprid-treated cells were respectively sealed, and 90.0 ± 1.7% and 85.0 ± 1.7% of control and imidacloprid-treated larvae respectively emerged as adults.

#### Short-term memory

Bees exhibited overall learning (significant trial effect: *F*_4,830_ = 2.49, *P* = 0.04). As shown in Fig. 1E, there was a significant interaction (*F*_4,830_ = 6.45, *P* < 0.0001) because learning increased in control bees (LSM contrast _t1 vs. t5_: *F*_1,830_ = 11.52, *P* < 0.0001) but not in imidacloprid-treated bees (LSM contrast _t1 vs. t5_: *F*_1,830_ = 0.99, *P* = 0.32).

Both control and imidacloprid-treated bees showed an increasing learning trend up to t3 (Fig. 1E). However, imidacloprid-treated bees exhibited poorer learning than controls in t4 (LSM contrast: *F*_1,287_ = 9.31, *P* = 0.003) and t5 (LSM contrast: *F*_1,287_ = 15.20, *P* < 0.0001). Control bees respectively exhibited 2.5- and 4.8-fold higher learning acquisition than imidacloprid-treated bees. Because imidacloprid effects only manifested in t4-5, there was no overall significant effect of treatment (*F*_1,287_ = 0.72, *P* = 0.40). Colony and bee identity respectively accounted for 0.9% and 11.3% of model variance.

Control (LSM contrast _t5 vs. t6_: *F*_1,1364_ = 12.96, *P* = 0.0003) and imidacloprid (LSM contrast _t5 vs. t6_: *F*_1,1364_ = 54.48, *P* < 0.0001) treated bees had improved longer-term as compared to short-term memory (1.7- and 7.8-fold increases, respectively). Thus, bees fed imidacloprid as larvae were deficient in short-term learning, but longer-term memory formation was not impaired.

#### Longer-term memory

The memories of treated larvae tested as adults exhibited nearly significant extinction over the tested times (trial effect: *F*_2,474_ = 2.82, *P* = 0.06, Fig. 1F). There was no significant effect of treatment (*F*_1,254_ = 0.08, *P* = 0.77). There was no significant interaction of trial*treatment (*F*_2,474_ = 0.22, *P* = 0.80). Colony identity and individual bee respectively accounted for 0.3% and 5.6% of model variance.

## Discussion

Imidacloprid is a widely used neonicotinoid pesticide throughout China^16^, but no studies have previously examined its effect on olfactory learning, a key element in successful foraging, for an economically^12^ and ecologically important^11^ native bee species, *A. cerana*. In adult bees, we show that ingestion of 0.1 or 1 ng/bee reduced olfactory learning acquisition, which was 1.6-fold higher in control bees. Effects of imidacloprid exposure during honey bee larval development are even less well understood. We provide the first evidence that *A. cerana* larvae exposed to imidacloprid (0.24 ng/bee) had significantly impaired olfactory learning as adults: control bees exhibited 2.5- and 4.8-fold better short-term learning acquisition. Our results support research suggesting that *A. cerana* may be more sensitive to pesticides than *A. mellifera*^13^,^14^. Giving 0.12 ng/bee did not impair olfactory learning in *A. mellifera*^42^, but a 0.1 ng/bee dose significantly reduced olfactory learning in *A. cerana*.

Short-term learning acquisition of control bees treated as brood was lower than for control bees treated as adults, but this may not be surprising. Newly emerged *A. mellifera* workers exhibit poorer olfactory learning than older workers^43^. Although we tested our larval-treated bees at 7 days of adult age, they were younger than adult-treated bees, which were collected from the nest entrance.

An alternative explanation for lower short-term PER learning of larval-treated bees is that control larvae were also exposed to imidacloprid. To control for potential colony differences, both groups were reared in the same colonies with imidacloprid-treated larvae. However, potential cross contamination between control and pesticide-treated larvae was limited. We added imidacloprid to brood food, which is not consumed by nurse bees. In feeding larvae, nurse bees could have come into contact with diluted imidacloprid and subsequently contaminated other brood, but these trace amounts would have been further diluted by the large number of brood in each colony. Moreover, even if there was cross-contamination, control group bees still showed far better (4.8-fold higher) learning acquisition than imidacloprid-treated group bees in the last reinforced learning trial (t5, Fig. 1E).

We used a brief 2 s exposure to CO_2_ (instead of cold exposure) to anesthetize our bees before harnessing (methods of Tan *et al.*^40^). Prolonged exposure to CO_2_ can reduce bee short-term learning^44^,^45^. Erber^45^ tested the effects of CO_2_ exposure duration on bee color learning and reported that CO_2_ narcosis takes about 1 min to impair memory. In contrast, we used a far shorter 2 s exposure. We also identically anesthetized control- and pesticide-treated bees. However, it is possible that CO_2_ narcosis altered the extent to which imidacloprid impaired shorter-term memory. This remains to be determined, though cold anesthesia also reduces bee olfactory learning^46^. CO_2_ narcosis likely did not affect our longer-term learning results because Kirkerud^44^ found no difference between longer-term learning of cold- or CO_2_-anesthesized bees.

### Larval exposure

Yang *et al.*^38^ showed that brood mortality increased when imidacloprid doses went from 24 to 8000 ng/larvae. They found no significant effects on brood capping, pupation, or eclosion at a dose of 0.4 ng/larvae^38^. We similarly found no effects of 0.24 ng/larvae on *A. cerana* brood capping or on survival to adult emergence. However, larval exposure to imidacloprid did impair subsequent adult learning. Thus, data on concentrations of xenobiotics in brood food would be valuable because little is known about what doses of neonicotinoids that larvae are exposed to. Wu *et al.*^41^ analyzed combs from *A. mellifera* colonies used for migratory beekeeping and found imidacloprid concentrations of 45 ng/g_comb_. Based upon typical imidacloprid concentrations found in sunflower pollen and nectar, Rortais *et al.*^47^ estimated that *A. mellifera* larvae would be exposed to 0.3 ng/bee over the first 5 days of development. Yang *et al.*^38^ demonstrated that an imidacloprid dose as low as 0.04 ng/larvae (given over the first four days of larval life) significantly reduced olfactory learning when *A. mellifera* workers were tested as 15-day old adults.

We administered imidacloprid over the first six days of larval life to *A. cerana* (total dose of 0.24 ng/bee) and then tested bees at 7 days of adult age. Although details of our PER assay (intertrial intervals and the total number of learning trials) differ from Yang *et al.*^38^, our overall results are similar. Control *A. mellifera* workers treated as larvae exhibited, on average, 2.0-2.4 higher PER learning acquisition than larvae exposed to 0.04 or 0.4 ng of imidacloprid in the last two learning trials^38^. In comparison, control *A. cerana* workers treated as larvae showed 1.7- and 7.8-fold higher PER learning acquisition in the last two learning trials (Fig. 1).

### Adult exposure

All of the imidacloprid concentrations and doses that we used in our study (20-100 μg/L) impaired short-term memory acquisition. Similarly, imidacloprid reduced *A. mellifera* learning acquisition when fed to bees at concentrations of 14.8-29.5 μg/L^30^,^39^ and 25.6 μg/L^34^ (values converted to μg/L for comparison). In *A. mellifera*, imidacloprid (59 μg/L) reduced short-term learning acquisition, decreased retention 1 h after the last learning trial, and increased the rate of memory extinction^48^. We show these same effects in *A. cerana* workers exposed as adults. Details of learning trial design, dosage, concentration, and whether bees have acute^30^,^39^ or chronic exposure^34^ matter, but our data follow a general trend. Imidacloprid over a wide range of sublethal doses can impair olfactory learning in at least two species of honey bees, *A. mellifera* and *A. cerana*.

### Summary

Olfactory learning plays a key role in foragers’ ability to return to rewarding food, and thus learning impairment may reduce colony fitness and health. Our results show that this impairment can affect adults and bees exposed as larvae. Effects may be more serious for bees exposed as adults. Bees exposed as adults continued to have impaired longer-term learning retention (retention was 1.3-1.8 fold higher in control bees), with both groups showing the same rates of memory extinction. Surprisingly, longer-term memory retention fully recovered after 1 h in bees that had received imidacloprid as larvae. In these bees, short-term learning shows an increasing trend up to t3, but then declined by 80% (t5) compared to controls (Fig. 1). Similarly, topical application of a neonicotinoid, thiamethoxam (0.1 ng/bee) to adult *A. mellifera* workers reduced olfactory learning tested at 24 h but not longer-term learning tested at 48 h^49^.

The reason our larval-treated bees recovered their longer-term memories but our adult-treated bees did not remains unclear. Adult-treated bees likely had far higher levels of imidacloprid and its metabolites in their bodies when they were learning and when they were tested than larval-treated bees, as a result of dosage and passage of time. We speculate that imidacloprid and its metabolites inhibited longer-term memory formation in adult-treated bees. Similar to imidacloprid (10-500 nmol/L), another neonicotinoid, clothianidin (1-100 nmol/L), and the imidacloprid metabolite, olefin, (50-500 nmol/L) block firing of mushroom body Kenyon cells and inhibit nicotinic responses, inactivating mushroom body neurons^50^. In larval-treated bees, imidacloprid may have selectively damaged neural pathways involved in short-term olfactory memory formation, and left longer-term memory formation relatively intact. However, little is known about the influence of imidacloprid on developing bee brains, a fascinating topic for future studies. In addition, further research on sublethal effects of neonicotinoids in other honey bee species and other bee species^5^,^51^ would be valuable, providing researchers and policy-makers a better sense of the impact of xenobiotics on important native pollinators.

## Methods

We used six *A. cerana cerana* colonies (three colonies per experiment) at Yunnan Agricultural University, Kunming, China from March through November of 2013.

### Imidacloprid concentrations and dosages

Imidacloprid can impair honey bee learning when applied topically^52^ or orally. We administered it orally because this is a likely exposure route. We fed bees imidacloprid (Haizheng Chemical Company, Taizhou, China) in 1.0 M sucrose solutions (30% sucrose w/w), and used the following doses: 0.24 ng/bee (fed to larvae over 6 days in 17.7 ppb solution = 78.2 nmol/L = 20 μg/L), 0.1 ng/bee (fed to adults in 8.9 ppb = 39.1 nmol/L = 10 μg/L), and 1 ng/bee (fed to adults in 88.7 ppb = 391.1 nmol/L = 100 μg/L). Lower doses correspond to imidacloprid levels that foragers could encounter while foraging. In the field, imidacloprid occurs at a maximum level of 912 ppb in pollen obtained from bee hives^9^. Imidacloprid residues occur at 1-50 ppb in nectar and pollen of a variety of crop species^21^. In citrus trees treated with imidacloprid, researchers measured residues of 3-39 μg/L in nectar^53^. Field realistic doses of imidacloprid from a variety of crops and studies are 0.7-10 μg/L, corresponding to a 0.024-0.3 ng dose per nectar load^54^. We used a 100 μg/L dose to determine if a higher dose would more strongly inhibit learning.

All doses were sublethal. In *A. mellifera*, only imidacloprid concentrations ≥1000 nmol/L increased mortality: 10 and 100 nmol/L did not alter mortality^34^. No *A. cerana* adult foragers given imidacloprid died during our experiments. There was also no significant effect of larval imidacloprid treatment on larval survival to emergence (see Results).

#### Experiment 1: Effect of imidacloprid on adult learning

We used 30 bees per colony per treatment (270 total bees). We captured likely foragers by approaching a colony gently to avoid arousing guard bees and using a clear plastic bottle to capture each bee as it flew away from the nest entrance (similar to the clear container capture method recommended by Matsumoto *et al.*^55^). We then anesthetized each bee by exposing it for only 2 s to a 100 ml/s flow of pure CO_2_ before harnessing it in a plastic tube^40^. Following standard protocol^56^, we discarded bees (<5%) that exhibited spontaneous PER to odor only or did not show PER after antennal stimulation with 1.0 M sucrose. Each bee was fed 10 μL of unscented, reagent-grade 1 M sucrose (30% sucrose w/w) containing one of three different treatments in: 0 ng (control), 0.1 ng (low-dose), or 1 ng (higher-dose) imidacloprid. We ran all three treatment groups in parallel during each trial.

We incubated bees (65% humidity, 25 °C) for 1 h after feeding to allow pesticide absorption and then placed bees in a conditioning apparatus in which they were familiarized with the main airflow (50 mL/s) for 15 s^39^. To present the conditioned stimulus (CS = hexanal odor) bees were exposed to a secondary air flow (2.5 mL/s) bearing a test odor, 10 μL of pure hexanal (Sigma-Aldrich, 98% pure, CAS# 66-25-1, Lot# MKBG1555V) pipetted onto a filter paper strip and inserted into a Pasteur pipette cartridge.

We used an olfactory conditioning protocol described by Bitterman *et al.*^56^. First the CS alone was presented for 3 s (proboscis extension scored during this time), and then the unconditioned stimulus (US = 30% pure sucrose solution containing no imidacloprid) was presented for 3 s. Both CS and US overlapped for 3 s in reinforced learning trials (Fig. 1A). To test short-term learning, we conducted five reinforced learning trials (t1-5), each with a 10 min intertrial interval. To test longer-term learning and memory extinction, we then tested bee responses to 3 s of odor alone with no sucrose reward (Fig. 1B) at 1 h (t6), 2 h (t7), and 11 h (t8) after the last reinforced learning trial (t5).

#### Experiment 2: Effect of larval exposure to imidacloprid upon subsequent adult learning.

We used three colonies to determine effects of larval exposure to imidacloprid. We obtained one brood comb from each colony and used a clear acetate sheet to mark where the queen had laid eggs. We then returned this comb to the colony. After eggs had hatched (3 days later), we removed the comb and gently injected 2 μL of 20 μg/L (78.2 nmol/L = 17.7 ppb) imidacloprid suspended in a 1 M sucrose solution into the brood food of each cell (which now contained a 1-day old larva). This treatment provided a daily dose of 0.04 ng dose/bee. We repeated this for 6 days, resulting in a total dose of 0.24 ng/bee during larval development. From each colony, we also obtained a separate brood comb as a control. We also fed larvae in the control comb for 6 days, but with a pure 1 M sucrose solution that contained no imidacloprid. Because differences in mortality from imidacloprid may be subtle, in each colony we treated 100 larvae with imidacloprid and 100 larvae with the sucrose control treatments but tested learning of only a randomly selected subset of these bees.

On the 7^th^ day (when *A. cerana* cells are normally sealed)^57^, we removed pesticide-treated and control combs, measured how many treated cells were sealed, and removed all eggs, larvae and pre-pupae that were not in control or pesticide-treatment groups. We then placed the combs in separate boxes in an incubator (35 °C at 60% humidity) for approximately 10 days until bees emerged. We counted the number of bees that emerged per treatment.

We kept newly emerged workers in the incubator with food naturally stored in their combs until they were 7 days of adult age. We then removed a random selection of bees, anesthetized them and harnessed them for PER testing as described above. Overall, <5% of these bees were excluded because they showed spontaneous PER to odor or failed to show PER upon antennal stimulation. Using the procedure described above, we tested 30 bees per treatment per colony. We used three colonies for a total of 180 bees.

### Statistics

We used JMP v11 Pro statistical software and report mean ± 1 standard deviation. For each experiment, we separately analyzed short-term learning acquisition (t1-5) and longer-term learning retention (t6-8, Fig. 1). We used repeated-measures, REML algorithm Analysis of Variance (ANOVA) to analyze the fixed effects of treatment (nominal variable) and trial (ordinal variable). Colony and bee identity were included in each model as random effects. To explore the detailed effects of treatment and trial, we performed a limited number of Least Squares Means (LSM) contrast tests. To compare longer-term memory retention with short-term memory acquisition, we compared t5 (last reinforced learning trial) with t6 (1 h after the first trial). We used chi-square tests to determine if treatment affected the number of cells that were capped and the number of larvae that survived to adulthood.

## Additional Information

**How to cite this article**: Tan, K. *et al.* A neonicotinoid impairs olfactory learning in Asian honey bees (*Apis cerana*) exposed as larvae or as adults. *Sci. Rep.* **5**, 10989; doi: 10.1038/srep10989 (2015).
